# Postmenopausal Osteoporosis: The Role of Immune System Cells

**DOI:** 10.1155/2013/575936

**Published:** 2013-05-23

**Authors:** Maria Felicia Faienza, Annamaria Ventura, Flaviana Marzano, Luciano Cavallo

**Affiliations:** ^1^Department of Biomedical Sciences and Human Oncology, University of Bari Aldo Moro, 70124 Bari, Italy; ^2^Institute for Biomedical Technologies, National Research Council, 70126 Bari, Italy

## Abstract

In the last years, new evidences of the relationship between immune system and bone have been accumulated both in animal models and in humans affected by bone disease, such as rheumatoid arthritis, bone metastasis, periodontitis, and osteoporosis. Osteoporosis is characterized by low bone mass and microarchitectural deterioration of bone tissue with a subsequent increase in bone fragility and susceptibility to fractures. The combined effects of estrogen deprivation and raising of FSH production occurring in menopause cause a marked stimulation of bone resorption and a rapid bone loss which is central for the onset of postmenopausal osteoporosis. This review focuses on the role of immune system in postmenopausal osteoporosis and on therapeutic strategies targeting osteoimmunology pathways.

## 1. Introduction

 In the last few years, there have been important advances in understanding the processes that regulate physiological and pathological bone turnover. 

Moreover, a relationship between the immune system and bone has long been speculated, as bone loss is a common condition of autoimmune and inflammatory disorders [1–3]. In this respect, T cells have been recognized as key regulators of osteoclast (OC) and osteoblast (OB) formation and activity in different diseases, such as rheumatoid arthritis [4], bone metastasis [5, 6], periodontitis [7, 8], congenital adrenal hyperplasia (CAH) [9–11], and osteoporosis [12]. 

In this review we focus on the involvement of immune system in the pathogenesis of osteoporosis with particular regard to postmenopausal osteoporosis and on the new therapeutic advances in its treatment.

## 2. Osteoporosis

To maintain a structural integrity, the skeleton needs to constantly remodel and repair the microcracks that develop both in cancellous bone, the “spongy” bone present in the vertebrae, pelvis, and metaphyses of long bones, and in cortical bone, the “compact” bone present in the diaphysis of the long bones and surrounding the cancellous bone in the vertebrae and pelvis. 

Osteoporosis is a systemic skeletal disease characterized by low bone mass and microarchitectural deterioration of bone tissue with a subsequent increase in bone fragility and susceptibility to fractures [13, 14].

*Skeletal fragility can result from* failure to produce a skeleton of optimal mass and strength during growth; excessive bone resorption resulting in decreased bone mass and microarchitectural deterioration of the skeleton; or inadequate response to increased resorption during bone remodeling [15].

The process of bone remodeling occurs in basic multicellular units (BMUs) which include OCs, OBs, and osteocytes and begins with the activation of hematopoietic precursors to become OCs, which normally requires an interaction with cells of the OB lineage. 

OCs are members of the monocyte-macrophage family and are derived from the fusion of marrow-derived mononuclear phagocyte, the OC precursors (OCPs), which circulate in peripheral blood (PB) [16]. These cells differentiate under the influence of two cytokines, namely, macrophage colony stimulating factor (M-CSF) and receptor activator of nuclear factor k-B ligand (RANKL). RANKL expressed on OBs and stromal cells as a membrane-bound protein and cleaved into a soluble molecule (sRANKL) by metalloproteinase [17] promotes differentiation and fusion of OCPs and activates mature OCs to reabsorb bone by binding to its specific receptor RANK. Osteoprotegerin (OPG), a soluble decoy receptor secreted by OBs and bone marrow stromal cells, competes with RANK in binding to RANKL, preventing its osteoclastogenic effect [17].

Mature multinucleated bone resorbing OCs are recognized by the expression of key OC markers including TRAP [18], calcitonin receptors [19], cathepsin K [20], pp60c-src [21], matrix metalloproteinase 9 (MMP9) [22], and the alpha V beta 3 integrin chains [23, 24].

Because the resorption and reversal phases of bone remodeling are short and the period required for OB replacement of the bone is long, any increase in the rate of bone remodeling will result in a loss of bone mass [15]. Moreover, the larger number of unfilled Howship's lacunae and Haversian canals will weaken the bone, and excessive resorption can also result in complete loss of trabecular structures, preventing bone formation.

Aside from postmenopausal osteoporosis which affects 30% of woman, there are many causes of secondary osteoporosis which occurs in almost 30–60% of men and more than 50% of premenopausal women [25]. Osteoporosis in children may be primary due to an intrinsic bone abnormality (usually genetic in origin) or secondary due to an underlying medical condition and/or its treatment. The most common condition in the former category is osteogenesis imperfecta in which there is an underlying abnormality in bone matrix composition, usually due to defective synthesis of type I collagen. 

Instead, osteoporosis circumscripta, characterized by focal osteolytic lesions [26], is a peculiar condition of Paget's disease, a skeletal disorder which affects 1-2% of adults over 50 [27, 28].

The evaluation of subjects presenting with osteoporosis should include a detailed history, physical exam, and laboratory testing for secondary causes of osteoporosis, according to the guidelines of the American Association of Clinical Endocrinologists (AACE) [29]. 

## 3. Postmenopausal Osteoporosis

The decline of ovarian function at menopause results in decreased production of estrogen and a parallel increase in FSH levels. The combined effects of estrogen deprivation and raising FSH production cause a marked stimulation of bone resorption and a period of rapid bone loss which is central for the onset of postmenopausal osteoporosis [30]. Several risk factors are implicated in favoring postmenopausal bone loss. Important nonmodifiable predictors of bone demineralization are age, sex, period of amenorrhea [31, 32], and parental history of fracture [33]. Important modifiable factors are dietary calcium intake [34, 35], low body mass index [31, 36, 37], smoking [38–40], reduced physical activity [41, 42], and high alcohol intake [43].

### 3.1. Estrogen Effects on Bone Remodeling

Estrogen is the major hormonal regulators of bone metabolism in women and men. Estrogen inhibits the activation of bone remodeling, most likely via the osteocytes, and also inhibits bone resorption, largely by direct actions on OCs, but also by modulation of OB/osteocyte and T-cell regulation of OC formation and activity [44].

The direct effects of estrogen on OCs include the induction of OC apoptosis and the inhibition of OC formation. In particular, this hormone inhibits OC formation decreasing the responsiveness of OCPs to the osteoclastogenic cytokine RANKL [45]. Moreover, estrogen inhibits RANKL-stimulated osteoclastic differentiation of human monocytes by inducing estrogen receptor *α* (ER*α*) binding to a scaffolding protein, BCAR1; the ER*α*/BCAR1 complex then sequesters TNF receptor-associated factor 6 (TRAF6), leading to decreased activation of NF-*κ*B and impaired RANKL-induced osteoclastogenesis [46]. 

In addition to these direct effects on OCs, estrogen also appears to regulate OC formation and activity indirectly. Combined in vitro and in vivo studies have demonstrated that estrogen suppresses RANKL production by OBs and T and B cells [47] and also increases production of the decoy receptor for RANKL, OPG [48]. 

In mouse models, estrogen modulates the production of a number of bone-resorbing cytokines, including interleukin (IL)-1, IL-6, tumor necrosis factor-*α* (TNF-*α*), M-CSF, and prostaglandins [49–53]. Thus, this indirect pathway may play a more important role in regulating the effect of estrogen on OC development, and estrogen deficiency induces bone loss by upregulating cytokine production in immune cells [54].

Regarding the role of estrogen on OBs, it has been demonstrated that they inhibit OB apoptosis and increase OB lifespan [55].

### 3.2. Estrogen-Deficiency Effects on Bone Remodeling: The Role of Immune System

Estrogen deficiency increases OC formation by increasing haematopoietic progenitors and providing a larger recruited OCP pool [56–58]. The upregulated formation and activation of OCs lead to cortical porosity and enlarged resorption areas in trabecular surfaces [13, 14]. In addition, estrogen depletion also increases the life span of OCs, and this event leads to prolonged bone loss, deeper resorption cavities, and trabecular perforation increasing the fragility of bone. This event contributes to a longer and slower period of bone wasting following acute phase of bone loss [14]. The bone loss is partly compensated by increase of bone formation due to the increased osteoblastogenesis. This event is fueled by increasing the number of mesenchymal progenitors capable of committing to the OB lineage and thus promotes proliferation of early OB precursors [56, 59, 60]. The net increase of bone formation, however, is limited by increasing apoptosis of OBs induced by estrogen deprivation [55, 61]. Therefore, although estrogen deficiency increases the bone remodeling intensity, there is an imbalance between bone resorption and bone formation [15, 62]. However, the mechanism by which estrogen deficiency induces bone loss seems more complicated, and interplay between estrogen deficiency and immune cells may play a pivotal role in regulating bone absorption in postmenopausal osteoporosis. In fact, estrogen is a well-known regulator of the immune system and T-cell functions [63, 64].

In this respect, studies on humans are few, and the majority of the data have been derived from animal models and cellular cultures, but no consensual picture has emerged from these models.

In one of the most interesting studies surface RANKL expression was quantified by two-color flow cytometry on isolated bone marrow mononuclear cells derived from premenopausal women, early postmenopausal women, and age-matched, estrogen-treated postmenopausal women. The surface concentration of RANKL per cell was increased in postmenopausal women compared to premenopausal women and estrogen-treated postmenopausal women by two- to threefold for MSCs, T cells, B cells, and total RANKL expressing cells [47]. This study suggests that osteoclastogenic RANKL production by T cells and B cells may contribute to bone loss during estrogen deficiency in humans, and it is supported by a recent work on mouse model [65].

Another clinical study underlines the role of T cells in the human postmenopausal bone loss.

This work reported that women with postmenopausal osteoporosis exhibit an increased T-cell activity and elevated production of TNF*α* and RANKL compared to healthy postmenopausal controls inducing OC formation and activity [12]. In particular, flow cytometry showed a higher percentage of OC precursors (CD14+/CD11b+/VNR+cells) from peripheral blood mononuclear cells (PBMCs) of postmenopausal women with osteoporosis than in the control groups. The mean fluorescence intensity (MFI) of CD11b and VNR was higher in patients than in samples from the control groups, while the MFI of CD14 was higher in the premenopausal controls and inversely correlated with age. This finding suggests that OCPs in patients were more committed toward osteoclastic lineage as compared to controls [12].

Recent clinical studies reported that postmenopausal women had a significantly higher concentration of circulating sclerostin than premenopausal women, and that serum sclerostin levels were inversely correlated with the free estrogen index in postmenopausal women [66]. In vivo and in vitro studies suggest that TNF-*α*, which is increased in estrogen deficiency, may stimulate the expression of sclerostin via the MEF2 transcription factor. Thus, the increase of sclerostin mediated by TNF-*α* may at least partially contribute to the pathogenesis of postmenopausal osteoporosis [67].

A recent study provides evidence that IL-17—a member of Th17 cytokine—promotes bone loss by favoring OC production and inhibiting OB differentiation, whose production is under the negative regulation of estrogen [68]. Moreover, an inhibition of IL-17 having bone sparing effect under ovariectomy by antibody approach could form the basis for using humanized antibody against this cytokine towards the treatment of postmenopausal osteoporosis [68].

### 3.3. B Lymphocyte Alterations in Postmenopausal Osteoporosis

B-cell alterations are well documented during aging and estrogen deficiency, but less is known on B lymphocyte status during osteoporosis. Recently, increasing evidence emerged on an intimate link between B lymphocytes and bone metabolism [69].

Although all women experience menopause and estrogen deficiency, only one third of them suffer from osteoporosis. In a recent study, Breuil et al. studied the phenotypic and functional characteristics of immune cells of 26 postmenopausal women with osteoporotic fractures compared to 24 healthy controls similar for age and estrogen level [54]. They observed, for the first time, a reduction of B lymphocyte number (in particular: B lymphocytes (CD19+), memory B lymphocytes (CD19+/CD27+), memory B lymphocytes expressing CD38 (CD19+/CD27+/CD5−/CD38+), and RANK+ memory B (CD19+/CD27+/RANK+) lymphocytes) in osteoporotic women negatively correlated with BMD. The authors postulated that this modifications of B-cell populations in osteoporotic women are the consequences of the physical changes, which took place in the bone marrow microenvironment, independently from age and estrogen status. Moreover, as memory B lymphocytes play a major role in the immune response to infections, the modifications of B lymphocytes may partly contribute to the increased morbidity and mortality observed after OP fracture [70].

## 4. Therapeutic Strategies of Postmenopausal Osteoporosis

The treatment of osteoporosis aims to reduce the incidence of vertebral and nonvertebral fractures responsible for the disease-associated morbidity [71] and stabilize or increase bone mass and strength [72].

The two main pharmacological approaches to osteoporosis are the anticatabolic and anabolic therapy, which, respectively, decrease bone resorption [73] and stimulate new bone formation [74].

The anticatabolic agents comprise bisphosphonates: etidronate, alendronate, risedronate, and zoledronic acid; estrogen and the selective estrogen receptor modulator (SERM) raloxifene; salmon calcitonin; and denosumab. The only anabolic agent currently available is teriparatide [75]. The treatment with bisphosphonates reduces fracture risk, not shown for other available agents. Bisphosphonates accumulate in the mineral phase of bone and reduce OC activity by inhibiting farnesyl pyrophosphate synthase [76]. They can be administered orally (daily, weekly, or monthly) or iv (quarterly or yearly). Since their initial introduction in the United States in 1995, questions have been raised about their association with possible side effects (osteonecrosis of the jaw, musculoskeletal pain, atrial fibrillation, atypical fractures, and esophageal cancer) that appear to be rare and may not be causally related [77]. However, for most patients with osteoporosis, the benefits of treatment outweigh the risks. A new therapeutic advance in the treatment of osteoporosis is denosumab, a fully human monoclonal antibody to soluble RANKL [78]. Denosumab is the newest antiresorptive agent, with a novel mechanism of action [79]. It acts like OPG, preventing RANKL from binding to OC receptor RANK; as a result, OC recruitment, maturation, and action are inhibited and bone resorption decreases. Unlike bisphosphonates, denosumab does not accumulate in bone. It has a circulatory half-life of approximately 26 days, and like other monoclonal antibodies, the clearance of denosumab is through the reticuloendothelial system and does not depend on renal clearance [80].

## 5. Conclusions

In the last years, many studies has been made to understand how the immune system impacts and regulates the skeleton in physiological and pathological conditions through the immunoskeletal interface. Although the majority of data derived from studies on animal models, recently new evidence of the crosstalk between immune system and bone has been accumulated in humans in many disease such as postmenopausal osteoporosis.

These data demonstrate that bone loss induced by estrogen deficiency in menopause is a complex effect of a multitude of pathways and cytokines working in a cooperative fashion to regulate osteoclastogenesis and osteoblastogenesis. Among these cytokines, RANKL and TNF*α* seem to play a central role inducing OC formation and activity, while IL-17 promotes bone loss by favoring OC production and inhibiting OB differentiation.

These discoveries have potential for developing new therapeutic strategies for the treatment of these bone disorders. In this respect, denosumab, a fully human monoclonal antibody to soluble RANKL, represents a new therapeutic advance in the treatment of osteoporosis with a novel mechanism of action that leads to the decrease of bone resorption and fracture risk.
